# Exploring the integration of dentistry within a multidisciplinary palliative care team: does bedside dental care improve quality of life and symptom burden in inpatient palliative care patients?

**DOI:** 10.1007/s00520-024-08671-4

**Published:** 2024-07-08

**Authors:** Sarah Uhlig, Florian Doberschütz, Franziska Hallmann, Hanna Salm, Jörg M. Sigle, Daniel Pink

**Affiliations:** 1 Klinik und Poliklinik für Innere Medizin C, Universitätsmedizin Greifswald, 17489 Greifswald, Germany; 2 Sarkomzentrum Berlin-Brandenburg, Helios Klinikum Bad Saarow, 15526 Bad Saarow, Germany; 3 Scientific IT Consulting, Riehen, Switzerland; 4 Zentrum für Zahn-, Mund- und Kieferheilkunde, Universitätsmedizin Greifswald, Greifswald, Germany

**Keywords:** Palliative care, Oral health, EORTC QLQ-C30, OH15, Quality of life, Patient reported outcome, Dental intervention

## Abstract

**Background:**

Despite the significant impact of oral problems on the quality of life of palliative care patients, comprehensive studies are lacking. This study is the first of its kind to address this gap by including both a dental examination and an intervention and assessing quality of life using the EORTC QLQ OH 15 questionnaire.

**Objectives:**

The objective of this study is to explore the impact of incorporating dentists into inpatient palliative care, with a focus on enhancing quality of life and alleviating symptom burden.

**Methods:**

In this monocentric study, data were gathered from a palliative care unit over an 8-month period. At the beginning of the multidisciplinary treatment, T0, patients underwent both a dental examination and interviews utilizing established questionnaires, the EORTC QLQ-C30 (core, general) and OH 15 (oral health). A week later, at T1, patients underwent a follow-up examination and interview. The QLQ-C30 and OH15 are widely recognized instruments developed by the European Organisation for Research and Treatment of Cancer (EORTC) for evaluating health related quality of life in cancer patients.

**Results:**

A total of *n* = 103 patients (48.5% women) were enrolled in the study. The median duration since their last dental visit was 1 year, and the dental condition at T0 was desolate. At T1, statistically and clinically significant changes in oral quality of life and symptom burden were observed. Noteworthy changes were noted in the OH-QoL score (median 63 vs. 92, *p* < 0.001), sticky saliva (median 33 vs. 0, *p* < 0.001), sensitivity to food and drink (median 33 vs. 0, *p* < 0.001), sore mouth (median 33 vs. 0, *p* > 0.001), and poorly fitting dentures (median 33 vs. 0 *p* < 0.001). Additionally, improvements were observed in xerostomia candidiasis and mucositis.

**Conclusion:**

The study highlights the powerful contribution of integrating a dentist in inpatient palliative care. With very little dental effort and simple ward and bedside treatments, significant improvements in the oral symptom burden of critically ill palliative patients can be achieved. This contributes to improved care status, relief of distressing symptoms, and ultimately improved quality of life. The results strongly support the consideration of dental support as an integral part of palliative care units.

## Introduction

The primary goal of palliative care is to administer compassionate support during the final stages of life for patients who no longer respond to curative treatment. The focus is not on prolonging life, but rather on enhancing its quality and effectively managing and alleviating symptoms like pain [1]. Burdening symptoms can be caused not only by general medical problems, but also by oral conditions. Due to the typically significantly reduced general condition of palliative patients, independent oral hygiene becomes challenging [2]. Recent studies show that palliative care patients often suffer from radiation caries, periodontal problems, xerostomia, candidiasis and oral mucositis, especially after chemotherapy or radiotherapy [3]. Patients typically receive interdisciplinary care from physicians of various specialties, nurses, chaplains, social workers, psychologists and physiotherapists [4]. Unfortunately, dentists and dental hygienists are often absent from this multidisciplinary team, which means that oral conditions that are usually present before admission to the palliative care unit often go undiagnosed and untreated [5].

In a survey of palliative care patients, 86% reported at least one oral symptom, including dry mouth (83.7%), pain (40.8%) or oral difficulties (51%). Ill-fitting dentures and denture sores were common among denture wearers [6]. Another study found a prevalence of 60% to 88% for xerostomia in palliative care patients. Here, the physical, social and psychological consequences were pointed out, as patients are restricted in food intake, speech and swallowing [7].

Given the significant impact on patients, it is unfortunate that oral disease and oral health often play a subordinate role in palliative care practice, as shown in another interview study with home carers [8]. This is even though a randomised controlled trial has shown that even a simple mouthwash based on a tea solution of Salvia officinalis brings relief from oral complaints in palliative cancer treatment [9]. It has also been published that untreated discomfort can lead to loss of appetite, malnutrition and cachexia and significantly impair quality of life [10].

Another relevant study also found that the details of oral health-specific problems in multidisciplinary palliative care are still unclear [11]. As there is a paucity of published experience on this topic, especially from Germany, the aim of this study was to investigate whether the inclusion of a dentist in a multidisciplinary palliative care team providing comprehensive, genuine dental care has a positive effect on the oral health, oral symptom burden and quality of life of palliative care patients.

## Methods

### Study design and data collection

The study was conducted at the Helios Klinikum Bad Saarow after approval by the responsible ethics committee from March to October 2023. The study adopted a monocentric prospective design, involving the collection of data through dental examinations and patient interviews. The study dentist conducted examinations both at the onset of the palliative complex treatment (T0) and one week later (T1). The examination consisted of a dental baseline examination in which the number of natural teeth (possibly with fillings, crowns), the number of functioning teeth (replaced teeth, implants) and the number of missing teeth (non-replaced, missing or severely damaged teeth) were recorded. In addition, the periodontal screening index (PSI/grades 1–4) was diagnosed to assess the presence of periodontitis. Furthermore, xerostomia was documented using the LENT-SOMA scale (grades 1–4), and the oral cavity was examined and categorised according to candidiasis (grades 0–3). The RTOG/EORTC-Toxicity Criteria scale (grades 0–4) was used to categorise mucositis [12]. Moreover, an examination of the oral cavity was conducted to identify potential issues such as pressure sores caused by dentures, herpes, oral inflammations and aphthae. The assessment encompassed an evaluation of oral hygiene, the appropriateness of dentures and the documentation of any aesthetic concerns. In the same session, the subjective self-assessment of quality of life was evaluated utilizing the established EORTC QLQ-C30 and the oral health-specific EORTC QLQ-OH15.

Following the initial examination, personalized recommendations were provided to each patient with the goal of addressing oral concerns and managing oral diseases in a tailored, prompt and effective manner. The treatment plan also considered the individual preferences, needs and challenges of each patient.

All interventions were carefully limited to minimal measures aimed to enhancing the quality of life within the palliative context. The recommended medications were part of the routine care of palliative patients and were tailored to the individual patient’s conditions and needs. Patients also received guidance and support with daily oral care, interdental care, lip care and denture cleaning. The oral cavity was regularly moisturised several times a day by the dentist and nursing staff using gels, sprays or oils. With the patient’s consent, tartar and sharp edges on dentures and teeth were removed. In addition, loose teeth were extracted on request, and the dentures were professionally cleaned and polished. Crucial data for the study were collected retrospectively from patient files to document the disease history, treatment and medication. At T1, the same parameters were recorded as at the initial examination, and the assessment of quality of life was once again conducted using the EORTC QLQ-C30 and OH 15.

### Outcome measures

Modules of the EORTC QLQ are developed using standardised procedures, designed for optimal clarity in several languages and extensively psychometrically validated. The Quality of Life Questionnaire C30 (QLQ-C30) developed by the European Organization for Research and Treatment of Cancer (EORTC) assesses the Global health status, five functional scales (physical, role, emotional, cognitive, social) as well as nine common symptoms in cancer patients. The QLQ-C30 consists of 30 items that relate to the state of health and well-being with scores ranging from 0 to 100. Higher symptom scores indicate higher symptom burden; however, higher scores in the global health status and the functional scales imply a better functioning.

The OH 15 combines questions about pain in the gum area, bleeding gums, sore lips, problems with teeth, sores in the corners of the mouth, dry mouth, changes in taste sensation when eating or drinking and problems eating solid foods in the Oral Health Quality of Life score. In addition, further questions are asked about symptoms. These include problems with sore mouth, sticky saliva, tooth sensitivity and dentures. Finally, the patient is asked how well informed they are about oral issues.

The QLQ-C30 and OH15 questionnaires were administered digitally on the patient’s quality-of-life recorder using AnyQuest for Windows [13]. Patients completed the electronic questionnaires on a Microsoft Surface Go 3 tablet PC, with assistance from the study physician as needed [14]. In addition, two PDF documents were available immediately after each recording—corresponding to a completed questionnaire and a completed analysis with graphical representation. This facilitated the identification of certain symptoms and functional limitations and supported communication between doctor and patient as a clear presentation in the medical record [15].

### Participants

Patients admitted to the palliative care ward at the Helios Klinikum Bad Saarow between March 2023 and October 2023 were enrolled in the study.

All patients admitted to the palliative care ward were considered for inclusion in the study.

Following an initial discussion about the patient’s state of health, a collective decision involving the study dentist, senior palliative care physicians and the ward manager was made to determine the patient’s eligibility for participation. If the patient’s health condition was deemed to severe, they were excluded from the study.

Furthermore, participation required written consent. To prevent result distortion, patients experiencing multiple hospitalizations were not included in the study repeatedly.

**Fig.1:**
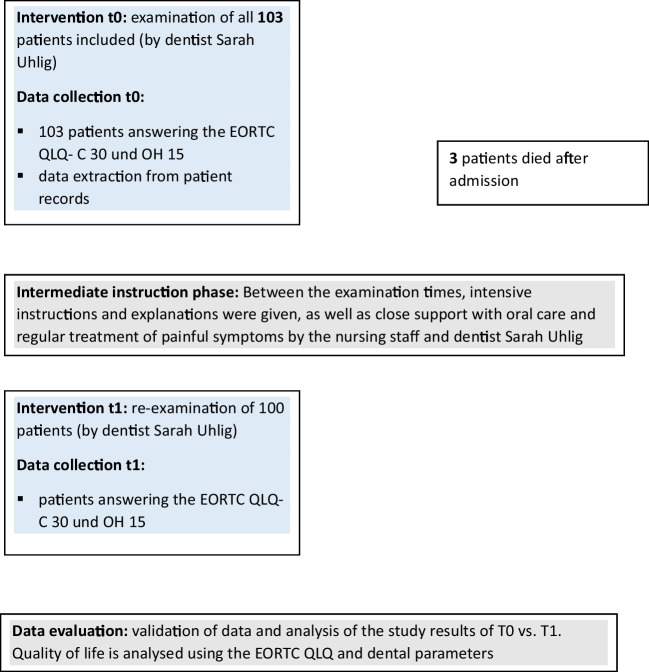
Flow chart for patient selection

The details of patient selection are shown in the flow chart below (Fig. 1). The details of patient selection are shown in the flowchart below (Fig. 1). Of course, all patients in the palliative care unit who were not included in the study for reasons such as lack of language skills, minority, transfer, poor general condition or refusal were offered dental care at any time after consultation with the attending physicians and the family.

### Statistical analysis

IBM SPSS Statistics 28 (SPSS Inc. an IBM Company, Chicago, IL) was used for the statistical analysis. Absolute and percentage frequencies were used to describe qualitative variables; quantitative or at least semi-quantitative variables were described using mean and standard deviation, minimum and maximum and median with 1st and 3rd quartiles.

The scales were tested for normal distribution using the Kolmogorov–Smirnov test. Due to significant deviations from a normal distribution, further statistical analysis was carried out using non-parametric methods. Thus, the non-parametric Wilcoxon sign ranks test was used to compare the calculated scale values from the QoL measurements at baseline (T0) and after one week (T1), i.e. before and after the intervention.

A two-tailed test with a significance level of 5% was performed. No adjustment was made for multiple testing, so the results were interpreted descriptively and exploratively.

### Ethical approval

The study was conducted after approval by the Ethics Committee of the Medical Association of Brandenburg (2022–204-BO-ff), the Ethics Committee of the Helios Klinik Bad Saarow.

(T-EXTERN-0003), and it was conducted in accordance with the Declaration of Helsinki.

### Founding sources

The study was conducted without financial backing or support.

## Results

### Patients

During the study period, 153 patients were admitted to the palliative care ward. A total of 103 patients were identified as being eligible to participate in the study. Three of these patients died before the second interview. Consequently, data from 103 patients were available at time T0; T1 data were available for 100 patients. Demographic dad for patients is shown in Table 1.

**Table 1:** Characteristics of the study population (*n* = 103)

Parameter	Group (*n* = 103)
Age (years) (mean ± SD)	72.14 ± 10.197
Gender (*n*, %)	
Male	53 (48.5%)
Female	50 (51.5%)
Malignancy	
Primary tumour in the body (*n*, %)	74 (71.8%)
Primary tumour in the head and neck (*n*, %)	18 (17.4%)
Haematological neoplasia (*n*, %)	7 (6.7%)
No underlying malignancy (*n*, %)	4 (3.8%)
Comorbidities (*n*, %)	97 (94.2%)
Active smokers (*n*, %)	17 (16.5%)
Taking more than 5 medications per day (*n*, %)	98 (95.1%)
Radiotherapy to the head and neck (*n*, %)	20 (19.4%)
Radiotherapy to the body (*n*, %)	52 (50.5%)
Chemotherapy (*n*, %)	67 (65%)
Bisphosphonates (*n*, %)	20 (19.4%)
Last visit at the dentist (years ± SD)	3.18 ± 5.424
Number of natural teeth (mean ± SD)	14.28 ± 10.454
Number of destroyed teeth (mean ± SD)	6.26 ± 8.738
Number of replace teeth (mean ± SD)	7.48 ± 10.450

Of these 103 patients, 50 were women (48.5%). The median age was 74 years. The patients’ underlying diseases were as follows: 74 patients had a solid malignancy with a primary tumour outside the head and neck (71.8%), 18 patients had a solid malignancy with a primary tumour in the head and neck (17.4%), 7 patients had a haematological neoplasia (6.7%), and 4 patients had other serious underlying diseases and no malignancy (3.8%).

Comorbidities were present in 97 (94.2%) patients; 17 (16.5%) patients were active smokers.

Ninety-eight (95.1%) patients were taking more than 5 medications per day. Twenty patients (19.4%) received radiotherapy to the head and neck, 52 patients (50.5%) received radiotherapy to the body, and 67 patients (65%) received chemotherapy. Bisphosphonates were also used in 20 patients (19.4%).

### Oral examination at T0

At the time of the first oral examination, the median time since the last visit to a dentist was one year. Five patients (4,9%) had no oral abnormalities and a good oral hygiene. These patients visited the dentist regularly, had adequate dentures and had no other oral symptoms.

On average, patients had 14 natural teeth; 7 destroyed or missing teeth not replaced by dentures; and 7 replaced teeth, including dentures, implants and pontics. The following table lists all oral symptom burdens, interventions, and caregiver availability at T0 (Table 2).

**Table 2:** Oral symptom burden at T0

Oral abnormality	Percentage	Oral intervention	Realisable by nursing stuff (yes/no)
Deficits in oral care	95.10%	Information on oral hygiene provided	Yes
Tartar	81.60%	Removed with scaler	No
Inflammation of oral cavity	67%	Regular rinsing with antiseptic	Yes
Rhagades	65%	Anointing lips and corners of mouth	Yes
Xerostomia	63%	Regular fertilisation	Yes
Insufficient dentures	48.50%	Denture adhesive	Yes
Cleaning of denture	47.60%	Cleaning with brush and gel	Yes
Sharp edges on denture	27.20%	Removed with sander	No
Loose or destroyed teeth	20.50%	Extraction recommended	Yes
Oral mucositis	17.40%	Anti-infective, antibiotics, dynexan, antifungals, benzydamine	Yes
Pressure mark on denture	13.90%	Removed with sander	No
Candidiasis	10.60%	Do not wear prosthesis and antifungal several times a day	Yes
Aphtha	8.70%	Ointment with local anaesthesia	Yes
Loose teeth with pain	3%	Extraction with anaesthesia	No
Herpes simplex	2.90%	Ointment with acyclovir	Yes

It should be emphasised that 84 patients (81.6%) had tartar, 50 patients (48.5%) had insufficient dentures and 98 patients (95.1%) had poor oral hygiene. In addition, 69 patients (67.7%) had inflammation in the oral cavity, and 21 patients (20.5%) had mobile teeth.

### Oral quality of life at T0

For the functional scale oral health-related symptom burden, the mean value at T0 was 60.60. For the symptom items, the mean score for sore mouth was 23.87, for sticky saliva 38.10, for sensitivity 35.52 and for dentures ill-fitting 38.63.

Only 4 patients had received information on oral hygiene from the treating physicians before the intervention. Only 3 patients were satisfied with the information they received.

### Interventions

All patients received educational and behavioural instructions as described under Methods. A check of all teeth and review of the oral cavity was performed on all patients. Further treatments were carried out on an individual basis and are shown in Table 2.

### Findings at T1

**Fig. 2:**
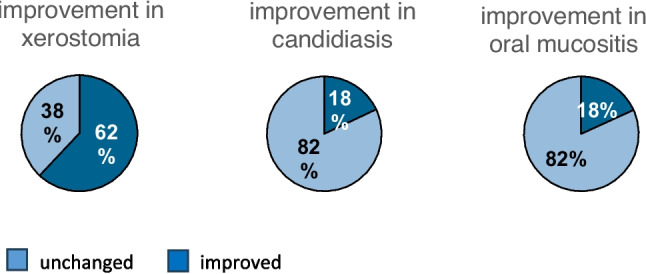
Xerostomia, candidiasis and oral mucositis after intervention

The large number of these measures had led to a significant improvement in subjectively perceived dry mouth at the time of the follow-up. (Fig. 2).

After the intervention, subjectively perceived dry mouth improved in 62% of patients. Treatment with an oral amphotericin solution or lozenges (Amphothericin B in the form of lozenge or as a suspension) also resulted in a significant improvement in the 16 patients (15.5%) who were diagnosed with candidiasis at baseline. At the second examination 7 days later, only 2 patients still had mild infestation.

Of 18 patients (17.4%) with mucositis at admission, including 10 patients with enanthema, 7 patients with focal mucositis and 1 patient with severe ulceration and pain who had been treated with lidocaine hydrochloride gel or mouthwash for pain relief, anti-infectives, antifungals and possibly antibiotics, only 3 patients had mild enanthema at the second visit. Oral hygiene improved significantly at T1. As a result, other oral symptoms such as stomatitis, rhagades, aphthae, herpes simplex and denture tenderness were virtually absent.

### Improvement in quality of life

In addition to the oral findings, general and specific oral health-related quality of life, as measured by the EORTC QLQ-C30 and OH15, improved as follows (see Table 3).

**Table 3:** Results of OH 15 Wilcoxon test for paired differences

	T0					T1					
	*N*	Mean	SD	IQR	Median	*N*	Mean	SD	IQR	Median	*p*
OH-Qol	103	60.60	23.668	33	63	100	88.40	13.734	17	92	** < 0.001**
OH-sticky saliva	103	38.10	36.619	67	33	100	11.28	21.792	33	0	** < 0.001**
OH-sensitivity	103	35.52	32.156	67	33	100	8.61	18.050	0	0	** < 0.001**
OH-sore mouth	103	23.87	31.462	33	33	100	4.98	15.958	0	0	** < 0.001**
OH-dentures ill- fitting	43	38.63	31.714	34	33	45	8.07	14.342	16.50	0	** < 0.001**
OH-information satisfied	3	44.33	50.954	100	33	100	94.72	12.159	0	100	0.180

**Fig. 3:**
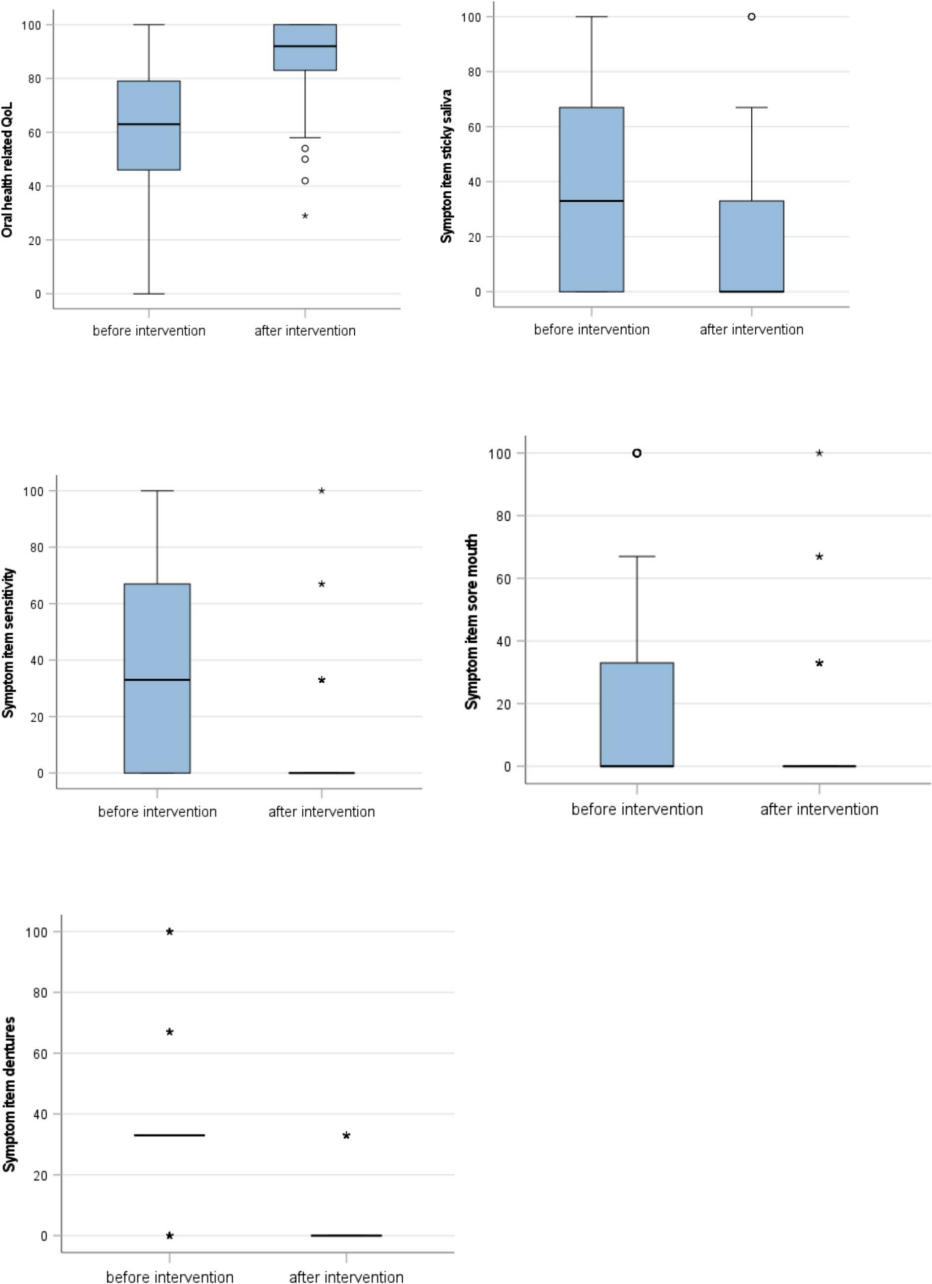
OH15 OH-Qol total score, sticky saliva, sensitivity, sore mouth and dentures before and after the intervention. *Wilcoxon test for paired differences, *p* < 0.001

At T1, statistically and clinically significant changes in oral quality of life and symptom burden were observed. This was the case for almost all scales of the OH15, including OH-QoL score (median 63 vs. 92 *p* < 0.001), sticky saliva (median 33 vs. 0 *p* < 0.001), sensitivity to food and drink (median 33 vs. 0 *p* < 0.001), sore mouth (median 33 vs. 0 *p* > 0.001) and poorly fitting dentures (median 33 vs. 0 *p* < 0.001) (Fig. 3).

Before the intervention, 4 patients had received information on oral hygiene from their dentist or physicians in other departments, but 96% of respondents denied this. After the intervention, almost all patients stated that they had received information.

## Discussion

The primary objective of this study was to elucidate the impact of dental intervention on patients’ QoL, explore shifts in oral symptoms and distress, and evaluate the efficacy of dental support.

### Oral deficits at T0

During the initial examination, it was evident that there was a concerning pattern of inadequate oral hygiene among the patients. Many had not visited a dentist for over a year.

The dental health status was distressingly poor, with patients showing oral pathologies, underscoring the requirement for more comprehensive dental care within palliative care settings. The pathologies encompassed dental calculus, infections of the oral cavity and oral rhagades. These findings are in line with results already presented by Singh et al. in India in 2021 and underscore the global relevance of the problem of inadequate oral healthcare in palliative patients [3].

Almost half of the dentures analysed in this study exhibited defects and were found to be unclean. Patients reported pressure sores and sharp edges. Due to severe weight loss, patients experienced dissatisfaction with the retention of their dentures. Difficulties with the dentures were previously documented by Guggenheimer et al. [16] citing xerostomia as the primary cause.

In addition to radiotherapy in the head and neck region, possible reasons for this include the use of medication that causes dry mouth or the combined use of several medications [17]. So 95.1% of the palliative patients taking part in the study were taking more than 5 different medications, which is essential for appropriate pain therapy. Severe dry mouth is considered a side effect of the therapy [9, 18].

In addition, many patients suffered from oral mucositis. This is in line with a study conducted by Pulito et al. (2020) citing that 90% of patients with chemotherapy and radiotherapy in the head and neck region suffer from this inflammatory reaction. This leads to a deterioration in quality of life, delays in further treatment, and a poorer prognosis [19]. According to Nicolatou-Galitis et al. (2001), additional infections often occur in this inflamed environment, which is also emphasised by our study [20]. Patients also suffered from candidiasis and herpes simplex.

Oral quality of life at T0 was assessed utilizing the EORTC QLQ-OH 15 questionnaire. The results of the multilevel scale for assessing oral health-related quality of life show an OH-Qol score (mean 60.60, SD 23.668). Compared to the study by Hjermstad et al. (2016), palliative patients rated their oral quality of life similarly poorly to cancer patients [21]. These results are also reflected in the symptom scores OH-sticky saliva (mean 38.10, SD 36.619), OH-sensitivity (mean 35.52, SD 32.156), OH-sore mouth (mean 23.87, SD 31.156), and OH-dentures ill-fitting (mean 38.63, SD 31.714).

### Results T1

Oral diseases and symptoms were found to improve in the presence of a dentist, notably in the successful treatment of xerostomia (improvement of 62%), candidiasis (improvement of 18%) and oral mucositis (improvement of 18%). These findings underscore the significant impact of dental intervention on enhancing oral health outcomes in palliative patients.

Furthermore, statistically significant improvements were achieved in the patients’ self-assessed health-related quality of life, OH-QoL (*p* < 0.001) and in symptom scales such as OH sore mouth (*p* < 0.001), OH sticky saliva (*p* < 0.001), OH sensitivity (*p* < 0.001) and OH information receives (*p* < 0.001), for which there is no other published experience to date.

### Feasibility

The dental examinations in the setting of this study could easily be carried out at the patients’ bedside or within their patient rooms. As already shown by Schimmel et al. (2007), most palliative patients are physically severely restricted, highlighting the recommendation of offering examination in a wheelchair or while the patient is in bed [22]. Patients frequently utilized their own hygiene products, a practice that proved beneficial as it allowed for the inspection and replacements of items as needed. All recommended medication and aids were conveniently kept on the bedside table, ensuring visibility for both caregivers and relatives regarding what needed to be utilized. It was easy to integrate the examination into the daily routine. Direct communication with the nursing staff and senior physicians proved to be beneficial for diagnosis and counselling. The importance of such collaboration is also emphasised by Malik et al., who assert the essential role of nurses in recognizing the necessity for oral care at the end of a person’s life [23].

The selected electronic questionnaires EORTC QLQ-C30 and OH15 on the QL-Recorder and circumscribed dental interventions were found to be feasible. In their large international study, Hjermstad et al. also came to the conclusion that the QLQ-C30 in combination with the OH15 module adequately records oral problems [21].

The OH15 module was chosen for its inclusion of symptom scales such as mouth ulcers, sticky or absent saliva, sensitivity and problems with dentures in addition to the functional oral health-related quality of life scale. Similar to the findings of Gorges et al., we conclude that the EORTC QLQ-OH15 module covers the most important topics despite the brevity of the questions and is well accepted by the patients [24]. Immediately after the survey, the results were recorded in the patient file and made available to the nursing staff. This enabled doctors and nursing staff to quickly gain an impression of the patient’s symptom burden and complaints.

### Strengths and limitations

A key limitation is that the sample was recruited exclusively from patients in a single institution. This limits the generalisability of the results to a broader patient population. Furthermore, many patients were excluded from the study due to the advanced stage of their disease, which further limits the representativeness of the sample. Another critical point is the short observation period of only seven days, without subsequent follow-up examinations, which does not take into account the long-term effect of dental interventions on the quality of life of palliative patients.

Despite these limitations, the study has significant strengths. A notable advantage is its potential reproducibility of the study, as it can be replicated in various clinical and outpatient settings. This reproducibility is crucial for validating the results and ensuring their applicability in different care contexts. In addition, the feasibility of the study requires little effort, as only one dentist needs to be involved. This enables a broad application of the study methodology and promotes understanding of the importance of dental care in palliative care. By repeating the study under varying conditions, the evidence base on the oral health of palliative care patients can also be systematically expanded and deepened.

### Implications for future research

The available data suggest that systematic dental care can significantly improve the oral health of palliative patients at the end of life. Despite this evidence, there is a lack of comprehensive studies looking at the implementation and impact of routine dental care in palliative care, as also highlighted by Walsh et al. in 2023 [25]. This research gap emphasises the need for further investigation to develop a deeper understanding of the specific needs and optimal treatment strategies for this patient group, as well as the importance of a palliative care guideline that provides clear treatment recommendations and support for typical oral cavity conditions. Although Jones et al. published such a guideline specifically for patients with progressive cancer in 2022. This guideline provides general information and emphasises the importance of oral care in palliative care [26].

The specific intervention by a dentist and the intervention and detailed recording of quality of life using the EORTC-QLQ OH 15 appears to be an innovative research approach and should be investigated further in the future. Such a concept is currently unique, as there are no comparable studies in the literature. In view of the novelty and significance of the results, further research perspectives open up that should aim to deepen the findings and promote the practical implementation of these interdisciplinary treatment approaches.

### Possible implementation in the clinic

The implementation of effective oral healthcare in clinical palliative care is a multidisciplinary challenge that extends beyond routine consultation with a dentist. A key strategy to improve the oral health of palliative care patients is the targeted training of caregivers, as suggested by Viebranz et al. [27]. This study emphasises the positive effects of individualised oral hygiene training on oral and denture hygiene in geriatric patients, highlighting the need to educate caregivers in the recognition and treatment of oral disease.

In addition, Shimotsu et al. (2024) point out specific problems such as candidiasis and emphasise the importance of regular inspections of the oral cavity by nursing staff. These measures are crucial to reduce the prevalence of oral pathologies and to initiate adequate therapeutic measures at an early stage, which both improve patients’ QoL and help prevent potential complications [28].

### Conclusion

The present research findings emphasise the need to consider oral health as an integral component of palliative care and to develop and implement appropriate care strategies. The identification of prevalent oral health problems underlines the urgency of integrative dental care within palliative care settings, aimed to enhancing the QoL for palliative patients and minimising the adverse effect of compromised oral health on overall health in the last phase of life. With very little dental effort and simple ward and bedside treatments, significant improvements in the oral symptom burden of critically ill palliative patients can be achieved. This contributes to improved care status, relief of distressing symptoms, and ultimately improved quality of life. The results of this study strongly support the consideration of dental support as an integral part of palliative care units.

## Data Availability

The data can be shared up on request.
